# Evaluating the Effect of Web-Based Iranian Diabetic Personal Health Record App on Self-Care Status and Clinical Indicators: Randomized Controlled Trial

**DOI:** 10.2196/medinform.6433

**Published:** 2016-10-21

**Authors:** Amirabbas Azizi, Robab Aboutorabi, Zahra Mazloum-Khorasani, Monavar Afzal-Aghaea, Hamed Tabesh, Mahmood Tara

**Affiliations:** ^1^ School of Paramedicine Department of Health Information Technology Ahvaz Jundishapur University of Medical Sciences Ahvaz Islamic Republic of Iran; ^2^ School of Medicine Endocrine Research Center, Metabolic Syndrome Research Center Mashhad University of Medical Sciences Mashhad Islamic Republic of Iran; ^3^ School of Health Management & Social Determinants of Health Research Center Mashhad University of Medical Sciences Mashhad Islamic Republic of Iran; ^4^ School of Medicine Department of Medical Informatics Mashhad University of Medical Sciences Mashhad Islamic Republic of Iran

**Keywords:** systematic review, diabetes mellitus, type 2, health records, personal, electronic health records, self care, Iran

## Abstract

**Background:**

There are 4 main types of chronic or noncommunicable diseases. Of these, diabetes is one of the major therapeutic concerns globally. Moreover, Iran is among the countries with the highest incidence of diabetic patients. Furthermore, library-based studies by researchers have shown that thus far no study has been carried out to evaluate the relationship between Web-based diabetic personal health records (DPHR) and self-care indicators in Iran.

**Objective:**

The objective of this study is to examine the effect of Web-based DPHR on self-care status of diabetic patients in an intervention group as compared with a control group.

**Methods:**

The effect of DPHR on self-care was assessed by using a randomized controlled trial (RCT) protocol for a 2-arm parallel group with a 1:1 allocation ratio. During a 4-month trial period, the control group benefited from the routine care; the intervention group additionally had access to the Web-based DPHR app besides routine care. During the trial, 2 time points at baseline and postintervention were used to evaluate the impact of the DPHR app. A sample size of 72 people was randomly and equally assigned to both the control and intervention groups. The primary outcome measure was the self-care status of the participants.

**Results:**

Test results showed that the self-care status in the intervention group in comparison with the control group had a significant difference. In addition, the dimensions of self-care, including normal values, changes trend, the last measured value, and the last time measured values had a significant difference while other dimensions had no significant difference. Furthermore, we found no correlation between Web-based DPHR system and covariates, including scores of weight, glycated hemoglobin (HbA1c), serum creatinine, high-density lipoprotein (HDL), low-density lipoprotein (LDL), total cholesterol, and planned visit adherence, as well as the change trend of mean for blood glucose and blood pressure.

**Conclusions:**

We found that as a result of the Web-based DPHR app, the self-care scores in the intervention group were significantly higher than those of the control group. In total, we found no correlation between the Web-based DPHR app and covariates, including planned visit adherence, HbA1c, serum creatinine, HDL, LDL, total cholesterol, weight, and the change trend of mean for blood glucose and blood pressure.

**ClinicalTrial:**

Iranian Registry of Clinical Trials (IRCT): 2013082914522N1; http://www.irct.ir/searchresult.php?id= 14522&number=1 (Archived by WebCite at http://www.webcitation.org/6cC4PCcau)

## Introduction

There are 4 main types of chronic or noncommunicable diseases (NCDs) [1]. Of these, diabetes is one of the major therapeutic concerns globally [2-5]. According to the World Health Organization (WHO) [6], diabetes is a chronic disease that occurs when the pancreas does not produce enough insulin or when the body cannot effectively use the insulin. Type 2 diabetes (formerly called noninsulin-dependent or adult-onset diabetes) is caused by ineffective use of insulin in the body.

In terms of improving the management of diabetes, efforts made to enhance the self-care status of diabetic patients are of utmost importance [7-9]. The management of chronic diseases such as diabetes mellitus (DM), in comparison with other chronic conditions, is heavily dependent on the individuals and regular assessment by the health care providers [10]. According to a report, approximately 90% of diabetics suffer from type 2 diabetes [11]. One of the most important concerns in the public health system is the medical care average cost of type 2 DM, which is almost 3 times more than others [12]. Therefore, improving self-care skills among individuals with chronic diseases will resolve many challenges to health systems.

Self-care behaviors refer to decisions a person can make and activities he or she can do to deal with a health issue or improve his or her health status. Self-care behaviors that people need to learn or improve in order to deal with type 2 diabetes effectively are self-monitoring of blood sugar, healthy diet, regular exercise, and adherence to medical treatment [13]. There are widely different models of self-care behaviors with the common feature in which the patient acts as the heart of health management. From the perspective of health promotion, health is taken into account as a source of daily life and self-care status is considered as empowerment. Thus, through the acquisition of self-care skills, people are able to actively get involved in decisions affecting their health [14].

It is recognized that the incidence of diabetes has been steadily rising for the past few decades around the world, especially with the highest rate growing more rapidly in middle- and low-income countries. According to the public call by WHO to cope with diabetes in all countries of the world together with its recognition as an alarm, especially in the developing countries, the management of such a disease by Iran’s Ministry of Health is taken into account as one of the research priorities [15,16]. Moreover, Iran is among the countries with the highest incidence of diabetic patients [17].

The reports presented by the International Diabetes Federation (IDF) classification cover the 20 countries and territories of the IDF MENA (Middle East and North Africa) region including Iran. There are over 37 million diabetics in these regions among the 387 million subjects suffering from this disease throughout the world and it is expected to reach 68 million by 2035. The number of such patients was 4.5 million in Iran in 2014, and in the same year, the incidence rate of the disease was 8.6% and 9% [18] in Iran and the world, respectively.

The therapeutic care of diabetic patients is still suboptimal despite international efforts often due to the lack of patient interactions with health care providers that are toward Web-based interventions [19]. Web-based personal health records (PHRs) are e-tools that allow patients to access health information via the Internet and take a more active role in their own health [20-22]. Patient-centric nature of PHRs make them ideal for patients to switch paternalistic model of medical care to a patient-centered model in which the patient is motivated to be an active and informed member of the health care team [23]. A review of the related literature on PHR revealed that such research studies have been different in terms of the following aspects:

- The first difference was associated with PHR format (paper or electronic). The review of literature indicated that the majority of research studies across the world have been conducted on electronic PHR. One significant reason could be that in such cases, requirements and prerequisites of electronic PHR studies are available, for example, an Electronic Health Record (EHR) or Electronic Medical Record (EMR) system is installed and PHR information is linked to that system.
- The second difference was associated with the subjects covered by PHR research studies. In other words, PHR has been conducted in multiple health issues, predominantly related to chronic diseases, including diabetes, cancer, and preventive care.
- The third difference concerned the sample size of PHR research studies. Some studies have been carried out on a small sample size [24,25] and others on a very large one [26]. In this respect, researchers found that paper-based PHR studies encompassed a small sample size and electronic PHR studies had been conducted by employing a large sample size.
- Last but not least, the fourth difference was linked with the study design of PHR research studies. The majority of studies in the field of PHR have been conducted in a retrospective manner. Additionally, there are numerous studies, merely library-based, discussing the definitions proposed for PHR. However, there are several studies evaluating PHR through quasi-experimental and randomized controlled trial (RCT).

There are numerous studies across the world, investigating the relationship between paper-based or electronic PHR and indicators such as self-care, self-efficacy, and quality of life as primary outcome measures. Moreover, in these studies, clinical indicators, including lipid profile, blood glucose, blood pressure, weight, and glycated hemoglobin (HbA1c) have been evaluated as secondary outcome measures [11,27-32]. Although there are many studies on the impact of PHR interventions on self-care index and clinical outcomes related to diabetic patients in the world, yet they are limited in developing countries. In addition, a similar study in Iran was conducted on the relationship between the use of paper-based diabetes follow-up card and self-management among diabetic patients [33]. Furthermore, library-based studies by researchers have shown that thus far no study has been carried out to appraise the relationship between Web-based DPHR and self-care indicators in Iran. According to previous studies, further research is needed to investigate the impact of electronic media on patient self-care behaviors [34,35]. The final DPHR model was systematically developed in our former study [36] and the purpose of this study is to evaluate the effect of the Web-based DPHR app on self-care status and clinical outcome measures. In this study, we hypothesized that the participants assigned to receive the Web-based DPHR app will manage better self-care compared with those who received usual care.

## Methods

### Study Overview

In the first phase, the initial version of the DPHR model was designed through systematic review and then validated and confirmed by the contribution of local endocrinologists. The details associated with the gray literature and databases, the quality appraisal of evidences, and the validation technique employed for DPHR through the Delphi method were mentioned in a review conducted by the authors of this study [36]. In addition, the details of the research method related to the second phase of this study are explained as follows:

#### Diabetic Personal Health Records App Development

Web-based DPHR app was coded through PHP programming language. Its server operating system was Linux and its database was MySQL. The app was developed by 2 professionals in this domain. In order to complete the DPHR development, 20 sessions were held for almost 200 hours. Two-type designed DPHR interface supported both the patients and the senior investigator (as a system administrator). SMS text messaging (short message service, SMS) and phone call were considered as reminders to check the DPHR system.

Web-based DPHR is a system by which type 2 diabetic patients can manage their health information associated with diabetes. The information in the app is obtained based on the systematic review of the valid references, including articles, reports, standards, and guidelines of international institutes. In sum, monitoring data, history of progress, appointment schedule, acquaintance with the disease; entering the health history, blood sugar levels, lab tests, blood pressure, weight, height and body mass index (BMI); and knowing the past and future time of medical advices and visits to improve self-awareness and self-care should be implemented easily through this app.

#### Usability Evaluation

Prior to the implementation of the app in a real context, the app interface was refined and optimized throughout the trial using heuristic usability evaluation techniques [37] by medical informaticians, endocrinologists, as well as using think-aloud technique by the participants. Moreover, a 3-part questionnaire was employed to elicit the views of the patients about the app. The components of the above-mentioned questionnaire were the general characteristics of the patients (including 10 data items), the user’s tasks (including 10 tasks), and the evaluation questions of the app (including 8 questions).

#### Functions of Diabetic Personal Health Records App

The functions of Web-based DPHR app are as follows:

- Identifying and maintaining a patient’s record: Through this function, users would be able to record and view the personal information, the urgent contact information, the diabetes information, comorbidities, the risk factors, and the allergy and vaccination information.
- Managing body mass index: Through this function, users would be able to record their weight and height, and subsequently body mass index is automatically calculated by the system.
- Managing lab tests: This function enables users to record their lab tests.
- Managing patient history: This function enables users to view all the information recorded and edit them if necessary.
- Managing patient visits: By employing this function, users would be able to view previous and future visits.
- Managing the physician’s advices: Through this function, users would be able to view the physician’s advices.
- Health dashboard: This section is one of the most important parts of the system by which the users could view the latest information in the form of graphs and view their status through the existing colors. For example, green represents normal status.

The research group and the app provider controlled development, updates, and maintenance of the DPHR system. The patients had additional interventions such as more visits and experimental tests, apart from prescribed therapeutic procedures of a physician to assess variations in the status of the health information.

### Study Design

The effects of DPHR on self-care status were assessed by using a RCT protocol for a 2-arm parallel group with a 1:1 allocation ratio. During a 4-month period, the control group benefited from the usual care; the intervention group additionally had access to the Web-based DPHR app besides routine care. Also, 2 time points at baseline and postintervention were used to evaluate the impact of the DPHR app.

The members who participated in the trial and gave informed consent based on some parameters including sex (male, female), employment status (employed and unemployed), and age ranges (≤30, 30-50, and ≥50 years), were randomly allocated in the 2 groups regarding covariate-adaptive randomization through SPSS version 21.0 (IBM Corp), by a person with no direct role in the research. A senior investigator and data analyst were blinded during the trial, unlike participants and practitioners who could not be blinded to DPHR since it was an obvious artifact. In addition, the individuals in both groups were not allowed to exchange DPHR information to avoid contamination of the trial.

### Participants

The statistical population of this 4-month trial in 2015 included patients suffering from type 2 DM in one of the endocrinology practice offices in Mashhad city, where there are over 120,000 patients with diabetes [38] considering inclusion and exclusion criteria. In the given office, there was a medical record for each patient in which all patient referrals were documented in its related record. In this study, we used the data from the records, including the number of patients based on their disease types, and extracted the demographic profile to do the introductory studies.

Only participants with signed informed consent were included in the study and were randomly divided into 2 groups: intervention and control. It should be noted that they manually received a package including a copy of the consent form, welcome letter, take-home manual, and stepwise instructions of the app usage. Their communication modes, in order to pose questions and concerns with the trial assistant, were either phone or SMS text messaging. The trained assistant required information concerning the intervention process, the Web-based DPHR app and the possible questions and answers.

The study’s inclusion criteria included: the age range of 20-70 years, resident of Mashhad, at least one-year history of having type 2 diabetes, knowledge of computers and access to the Internet, high school diploma or above, as well as completing an informed consent. The exclusion criteria included lack of cooperation or inability to perform the study for any reason such as sickness, pregnancy, immigration, and so on.

### Sample Size

There was no previous research about self-care among the Iranian population to estimate the sample size, except a survey showing an association between self-care activities and the quality of life [39]. Thus, in this work the sample size of 60, corresponding to the formula, was estimated based on the same one in Iran [40]. Finally, 72 patients in the 2 groups, of whom 36 cases were from the intervention group receiving the DPHR app and 36 from the control group with the routine cares, were enrolled according to the confidence interval of 95%, the power of 80%, and the dropout rate of 20%.

### Outcome Measures of Study

In this study there was one primary outcome and several secondary outcomes. To evaluate the self-care status as the primary outcome measure, a researcher-made questionnaire composed of 7 sections with independent items was developed. This questionnaire was adapted from the existing valid literature in the field of self-care [41-44]. Self-care is one of the measures related to knowledge used by patients and in fact, the reason for selection of this criterion [45]. The dimensions of the self-care questionnaire are as follows: general information (25 questions), information of normal values (10 questions), information of change trend (10 questions), information of physician advices (2 questions), visit information (3 questions), information of the latest measurement values (9 questions), information of date and time of the latest measurement values (9 questions), and information of training tips (13 questions). In addition, some secondary outcomes were observed in this study. Any potential relation compared with cause and effect was investigated between the use of the app and the diabetes follow-up clinical indicators as secondary outcome measures. Such indicators were: fasting blood sugar (FBS), 2-hour postprandial blood sugar, weight, blood pressure, lipid profile (total cholesterol, triglyceride, HDL, and LDL), HbA1c, and serum creatinine. Furthermore, another secondary outcome measure was the adherence of patient to planned visit. The outcome measures of this study is presented in Table 1.

**Table 1:** Outcome measures of study.

Outcome measures		Measurement time points		
		Baseline	Weekly	Postintervention
**Primary**				
	Self-care status	X		X
**Secondary**				
	Blood sugar: FBS 2-hour postprandial	X	X	X
	Weight	X		X
	Blood pressure: Systolic Diastolic	X	X	X
	Lipid profile: Total cholesterol Triglyceride HDL LDL	X		X
	HbA1c	X		X
	Serum creatinine	X		X
	Adherence to planned visit	X		X

### Data Collection

We began the study by acquiring the following baseline information: gender, age, marital status, occupation, education level, family history of DM, types of drug used, history of high blood pressure, access to home monitoring tools (glucometer, sphygmomanometer, scale), computer literacy, access to the Internet, working time with a computer, length of disease, FBS, 2-hour postprandial blood sugar, blood pressure, weight, lipid profile (total cholesterol, triglyceride, HDL, and LDL), serum creatinine, and HbA1c along with the information required to check the inclusion eligibility.

The trial assistant informed the patients elected by the inclusion criteria about the details of the study and then obtained informed written consent, as well as the tool required including the questionnaire that was completely anonymous to respect the ethical considerations. Each form had a unique code to manage further references. They were free to contact for sharing the questions and concerns by available communication means during the entire project. The assistant provided a username and password to access the DPHR app, and data were recorded securely only by authorized members of the trial team.

The face and content validity of the self-care questionnaire was assessed by the experts’ opinions (including 2 endocrinologists, 1 medical informatician, and 1 methodologist) and valid literature. Also, the questionnaires were completed through structured interviews by a trial assistant blinded in the study, and the participants completed the self-care questionnaire on paper in person at the diabetes clinic during the follow-up phase. The final score of the self-care index was obtained from the total correct responses for each item in the 7-part questionnaire. Each correct response was assigned a score of 1 and the wrong answers, a 0. The scores of each dimension of the questionnaire were obtained from the sum of correct answers to the items of that dimension.

**Figure 1:**
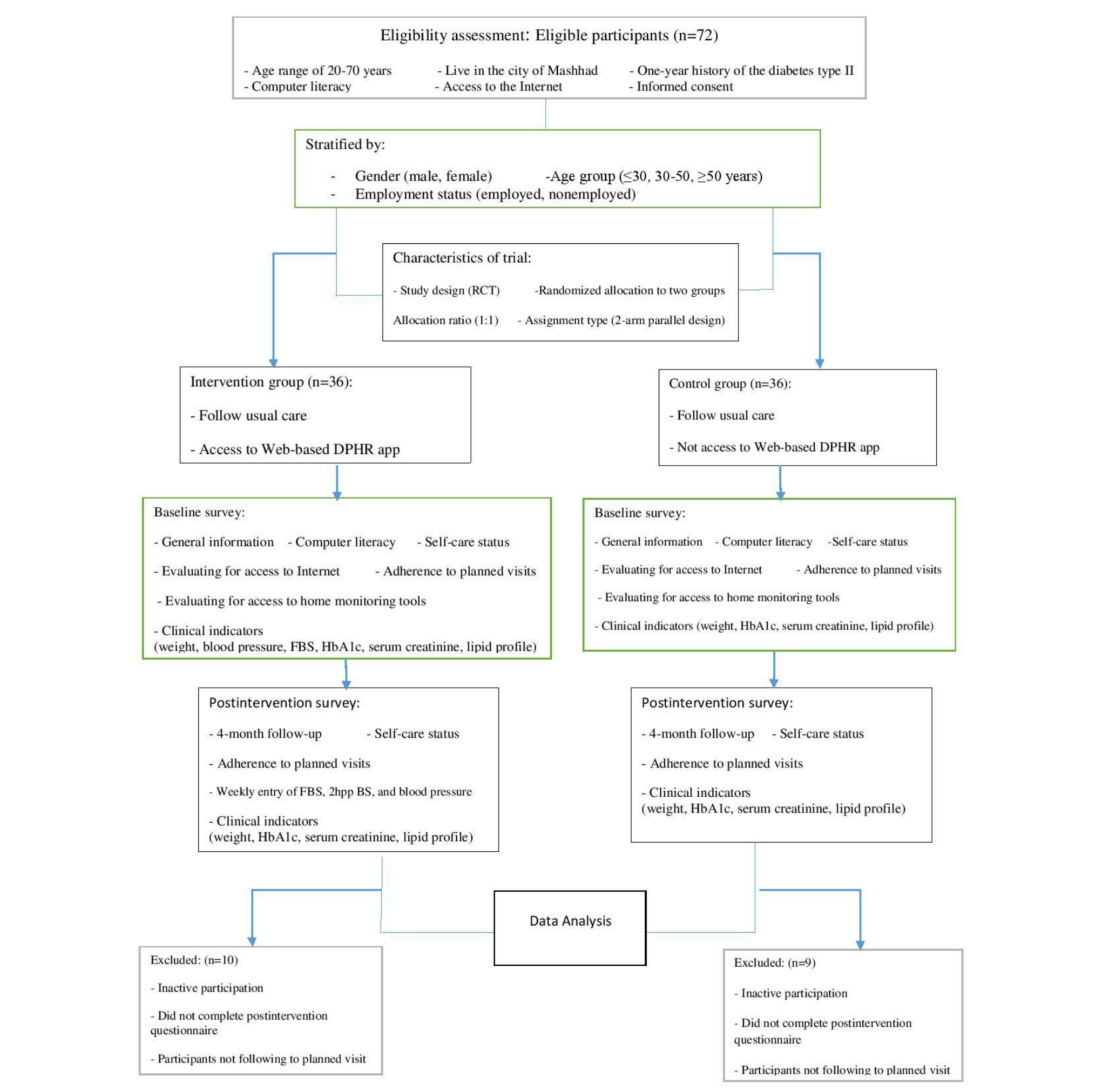
Flow diagram of study procedures. DPHR: diabetic personal health record; FBS: fasting blood sugar; Hb^A1c^: glycated hemoglobin; 2hpp BS: 2-hour post-prandial blood sugar.

Data related to HbA1c, lipid profile, and weight in pre- and post-intervention were gathered in both control and interventions groups, but weekly blood sugar and blood pressure measurements were done only in the intervention group where the patients were responsible for doing them on their own, including weight measures. Other tests such as HbA1c, lipid profile and serum creatinine were performed in a laboratory based on objective- and laboratory-based measures. Moreover, the reminder means to view the app or complete their self-care actions were through weekly SMS text messaging or phone call. Figure 1 depicts the flow diagram of the study procedures.

### Data Analysis

The statistical significance of the 2-tailed analyses in this study was performed with a significance interval of 95% and alpha level was set at *P*<.05. The data were analyzed through descriptive analysis (frequency, percent, and mean) and inferential analysis (normality test, paired and unpaired t-test, chi-square test, Fisher’s exact test, Mann-Whitney U test, and 1-way ANOVA), using SPSS version 21.0. The descriptive statistics analyzed distribution of variables, website usage statistics, and app visits. In case of analyzing the variables under review, initially the value of outcome measure (eg, self-care) was analyzed independently in both groups according to data of baseline and postintervention phases using the paired t-test analysis and then the scores of both groups were analyzed using the independent t-test analysis.

### Ethical Considerations

This study, registered on Iranian Registry of Clinical Trials (IRCT), received approval from the Research Review Committee and the Regional Ethics Committee (approval # 921835). Moreover, the details of research protocol have been published [46].

## Results

### App Usage

Statistics on DPHR app usage were obtained through webserver log analysis. The final analysis comprised 27 out of 36 participants in the control group and 26 out of 36 in the intervention group. Hence, the rates of patient response to the self-care questionnaire were 75% and 72% in the control and intervention groups, respectively.

According to Table 2, the maximum frequency of measurement record was 63 times, and the minimum value was 10 times. Blood glucose followed by blood pressure, weight, and lab tests were the most-recorded parameters during the study. In total, the highest record was related to blood sugar levels with a frequency of 450 times (mean 17.3), and the lowest one was associated with lab test entries with a frequency of 53 times (mean 2). Additionally, 38.5% (10/26) had recorded the measurement less than 20 times, 38.5% (10/26) 20-30 times, and 23% (6/26) over 30 times. The details related to the variables under review recorded by the patients in Web-based DPHR app are presented in Table 2.

**Table 2:** The frequency and average of trial variable entries in diabetic personal health records (DPHR) app (n=26).

Patient ID	Weight and height	Blood pressure	Lab tests	Blood sugar	Total
1	2	3	2	8	15
2	2	2	2	11	17
3	4	8	4	10	26
4	2	4	2	12	20
5	3	18	2	40	63
6	2	4	2	16	24
7	3	1	2	9	15
8	1	3	2	10	16
9	2	8	2	41	53
10	2	4	2	8	16
11	4	7	2	15	28
12	4	10	2	40	56
13	2	4	2	6	14
14	2	3	2	36	43
15	2	4	2	14	22
16	3	4	2	10	19
17	2	10	2	24	38
18	1	3	2	4	10
19	3	4	2	37	46
20	2	3	2	8	15
21	2	6	1	15	24
22	2	3	2	15	22
23	2	2	2	20	26
24	2	3	2	13	20
25	1	3	2	16	22
26	2	3	2	12	19
Total	59	127	53	450	689
Mean	2.2	4.8	2	17.3	26.5

**Table 3:** The demographic profile and opinions of diabetic patients participating in usability evaluation of diabetic personal health records (DPHR) app (n=6).

No.	Gender	Age (year)	Employment status	Education level	Time (minute)
1	Female	50	Unemployed	BSc	40
2	Female	58	Employed	BSc	20
3	Male	36	Employed	BSc	30
4	Male	38	Employed	BSc	25
5	Female	58	Unemployed	Diploma	20
6	Male	61	Unemployed	Diploma	45

**Table 4:** The opinions of experts and diabetic patients participating in usability evaluation of diabetic personal health records (DPHR) app.

Target groups	Opinions
Medical informatics students	Systolic blood pressure should be written in Persian.
	It would be better to use the full screen, especially in browsing the page in order not to scroll so much.
	The fonts of data entry forms were different from those of report forms.
	In the graphs of blood sugar history, the values could be shown in green or red for the normal and abnormal ranges in order to help patients know their status.
	Charts in the main page were incomprehensible.
	The first and the last names should not be entered in numeric characters.
	In the national code section, validation is required in order to enter valid national codes.
	The entry of non-numerical characters should be prevented as a patient number.
	There is a problem with the measurement turn: it is better to be entered by the system.
	It is better to set proper labels for each axis of the charts.
	Fonts in green are not good at all.
	Submit button is pale and blurred.
	Numeric default values have been defined in blank fields, while it is better to enter dashes if no values are entered.
	Mandatory fields are required to be marked with an asterisk.
Endocrinologists	Instead of insulin-dependent diabetes, type 1 diabetes must be used.
	In the diabetes treatment section, the term “others” should be deleted.
	In the comorbidities section, the term “cataract” should be deleted.
	In the neuropathy section, the term “behavioral disorders” should be deleted.
	In eye diseases, the term “glaucoma” should be added.
	The term “goiter” should be written in the form of “simple goiter.”
	The term “intermittent claudication” should be written instead of “ischemic pain of organs” and should be placed in the section of cardiovascular diseases.
	The time of blood glucose measurement needs to be determined.
Type 2 diabetic patients	Home icon should be used next to the term “homepage.”
	Blood glucose list numbers should be displayed.
	Instead of millimeters of Mercury, the unit of centimeters of Mercury should be used for hypertension.
	Abnormalities in the graph should be shown with a different color.
	The patient is required to read the guide.

**Table 5:** The demographic characteristics and distribution difference of participants in control and intervention groups.

Variable	Frequency (percent)		
	Control group	Intervention group	*P* value of distribution difference of variables in 2 groups
	(n=27)	(n=26)	
**Gender**			
Male	11 (41)	15 (58)	.28
Female	16 (59)	11 (42)	
**Age group (year)**			
≤30	1 (4)	0	.53
30-50	5 (18)	9 (35)	
≤50	21 (78)	17 (65)	
**Marital status**			
Single	3 (11)	0	.24
Married	24 (89)	26 (100)	
**Employment status**			
Employed	12 (44)	18 (69)	.01
Unemployed	15 (56)	8 (31)	
**Education level**			
Diploma	7 (26)	5 (19)	.38
Associate and BSc	17 (63)	16 (62)	
MSc and PhD	3 (11)	5 (19)	
**Family history of Diabetes Mellitus**			
Yes	21 (78)	20 (77)	>.99
No	6 (22)	6 (23)	
**Type of drug taken**			
Insulin	9 (33)	5 (19)	.85
Oral	11 (41)	17 (66)	
Insulin and oral	7 (26)	4 (15)	
**History of high blood pressure**			
Yes	17 (63)	15 (58)	.78
No	10 (37)	11 (42)	
**Access to measurement tools at home**			
Glucometer	6 (22)	3 (12)	.53
Glucometer, sphygmomanometer, scale	10 (37)	16 (61)	
Glucometer, sphygmomanometer	9 (33)	3 (12)	
Glucometer, scale	2 (8)	4 (15)	

### Usability Evaluation of Diabetic Personal Health Records App

To have a preliminary usability evaluation of DPHR app, the usability questionnaire was submitted to 20 PhD and 30 MSc students of medical informatics. Of them, 11 PhD and 8 MSc students responded. In addition, 1 medical informatics expert, 2 endocrinologists, and 6 type 2 diabetic patients contributed in this respect. The usability evaluation also lasted for almost 50 days.

To evaluate the usability of Web-based DPHR app by diabetic patients, 6 people, including 3 women and 3 men, participated in this study. In terms of level of education, 4 participants had BSc and 2 had high school diploma degrees. The mean age of the patients was 50 years, and the average time of evaluation sessions was 30 minutes. The details related to the demographic profile and the opinions of diabetic patients and experts participating in usability evaluation of DPHR app are indicated in Tables 3 and 4.

### Descriptive Analysis

The descriptive analysis of both intervention and control groups was conducted separately using frequency, percent, and mean for qualitative and quantitative variables. In the control group, there were 16 out of 36 women (59%) and 11 men (41%) with a mean age of 57 years. In terms of level of education, 17 out of 36 individuals (63%) held associate and BSc degrees. Considering employment status, 12 out of 36 participants (44%) were employed and 15 participants (56%) were unemployed. On an average, they worked with a computer for 8.5 hours per week.

In the intervention group, there were 11 out of 36 women (42%) and 15 men (58%) with a mean age of 52 years. In terms of level of education, 16 out of 36 individuals (62%) held associate and BSc degrees. Considering employment status, 18 out of 36 participants (69%) were employed. On average, they worked with a computer for 18 hours per week. Details relating to the demographic characteristics and distribution difference of participants in control and intervention groups are presented in Table 5.

### Confounder Analysis

To analyze the equality of distribution for some variables which were likely to be confounder variables, the chi-square test was applied to qualitative variables, including gender, age group (year), marital status, employment status, education level, family history of DM, types of drug taken, history of high blood pressure, access to measurement tools at home, computer literacy, range of working time with a computer (hour), and range of disease length (year). The test results demonstrated no significant differences in the distribution of the variables between the intervention and control groups other than the variable of the range of working time with a computer, where participants in the intervention group at the baseline stage had spent more time working with a computer (Table 4).

### Normality Analysis

To compare self-care indicators and their dimensions in the control and intervention groups, their normality was first evaluated using the Kolmogorov-Smirnov (K-S) test, which revealed that the distribution of self-care indicators was normal. Moreover, in the control group at the baseline stage, the dimensions of self-care status, including information of normal values, information of change trend, information of the latest measurement values, and information of training tips were normal in terms of distribution; however, other dimensions such as information of the physician’s advices, visit information, and information of date and time of the latest measurement values were abnormal.

Additionally, in the intervention group, the dimensions of self-care status including information of normal values, information of change trend, information of the latest measurement values, information of date and time of the latest measurement values, and information of training tips were normal in terms of distribution; however, other dimensions such as information of the physician’s advice and visit information were abnormal.

### Inferential Analysis

In continuation, the parametric tests such as independent T-test were employed for analyzing the distribution of self-care indicators and their dimensions with normal distribution in both groups at the baseline stage, and for dimensions with abnormal distribution, the nonparametric tests such as Mann-Whitney U test was applied. The test results indicated that distribution of self-care indicators and their dimensions other than the sixth dimension, that is, information of training tips, were not significantly different in both groups at the baseline stage.

To compare the scores of self-care indicators in both groups, the independent T-test was employed. Test results revealed that there was a significant difference in terms of self-care indicators in both groups of diabetic patients.

Moreover, we found a significant difference in the dimensions of self-care indicators, including information of normal values, information of the trend of change, information of the latest measurement values, and information of date and time of the latest measurement values. However, no difference was observed in other dimensions such as information of the physician’s advice, visit information, and information of the training tips.

In addition, the independent T-test was utilized to compare the scores of weight, HbA1c, serum creatinine, HDL, LDL, total cholesterol, and triglyceride in control and intervention groups. The test results revealed no significant difference between any of them. Details relating to the comparison of average difference of self-care indicator, its dimensions and clinical outcomes in control and intervention groups are outlined in Table 6.

**Table 6:** A comparison of the average difference of self-care status, its dimensions, and clinical outcomes in control and intervention groups.

Outcome measure	Dimensions	Mean (SD)			
		Control group (n=27)	Intervention group (n=26)	*P* value	95% CI
**Self-care status**	Information of normal values	1 (1)	2.8 (1)	<.001	(-2.3 to -1.1)
	Information of change trend	-0.2 (0.8)	1.3 (2)	<.001	(-2.4 to -0.6)
	Information of physicians’ advice	0.1 (0.4)	0.2 (0.4)	.73	(-0.2 to 0.2)
	Visit information	0.26 (0.447)	0.3 (0.5)	.94	(-0.2 to 0.2)
	Information of latest measurement values	0.04 (1)	1.9 (1.6)	<.001	(-2.6 to -1.1)
	Information of date and time of latest measurement values	-0.2 (1)	2 (1.5)	<.001	(-2.9 to -1.4)
	Information of training tips	1.7 (1)	2 (2.6)	.51	(-1.4 to 0.7)
	Self-care indicator	2.8 (2.4)	10.6 (4.5)	<.001	(-9.7 to -5.8)
**Clinical outcomes**	Weight	0.03 (1)	-0.9 (2.4)	.08	(-0.1 to 2.0)
	HbA1c	0.2 (0.1)	-0.2 (0.1)	.22	(-0.2 to 0.8)
	HDL	-0.4 (11)	-5 (17)	.298	(-4.6 to 14.6)
	LDL	4 (35)	-3 (23)	.44	(-10.8 to 24.8)
	Total cholesterol	-14 (57)	-6 (26)	.75	(-64.3 to 47.8)
	Triglyceride	-4.5 (157)	-26 (45)	.56	(-53.6 to 96.9)
	Serum creatinine	0.05 (0.3)	-0.01 (0.3)	.42	(-0.1 to 0.2)

**Table 7:** The comparison of visit adherence in control and intervention groups.

Visit adherence	Group		Total	*P* value
	Control	Intervention		
No	3	1	4	.61
Yes	24	25	49	
Total	27	26	53	

Fisher’s exact test was also used to compare visit adherence scores in both groups, and the test results showed no significant difference. Table 7 provides the comparison of visit adherence in control and intervention groups.

**Figure 2:**
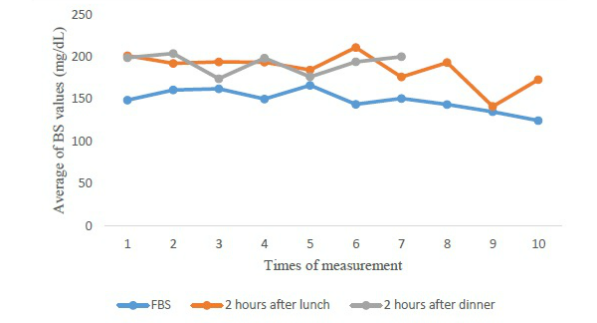
Change trend of mean related to blood sugar in intervention group. BS: blood sugar; FBS: fasting blood sugar.

The change trend of mean for FBS, 2 hours after lunch and 2 hours after dinner, indicates fluctuations in the 2-hour-after-dinner trend. However, the variations during the 2 hours after lunch had a steady state until the fifth measurement and then they had an unstable mode. Furthermore, FBS was almost in an unstable mode, although, a reducing pattern was observed in the final measurements. Figure 2 provides the change trend of mean related to blood sugar in intervention group.

**Figure 3:**
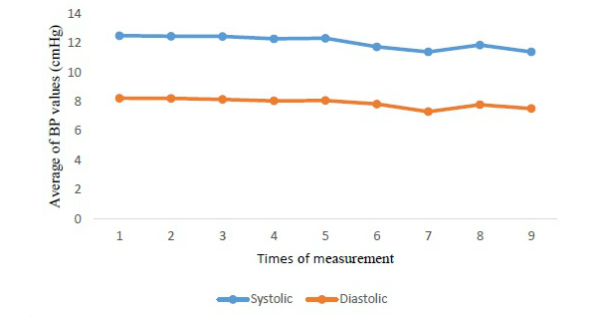
Change trend of mean related to blood pressure in intervention group. BP: blood pressure.

The mean trend of systolic and diastolic blood pressure demonstrates the steady and stable trends, and no increasing or decreasing patterns were observed. Figure 3 provides the change trend of mean related to blood pressure in intervention group.

The relationship between covariates and self-care indicators for the 2-state variables such as gender and employment status was analyzed through T-test. Furthermore, the 1-way analysis of variance (ANOVA) was applied to multi-state variables such as education level, age group, length of disease, computer literacy, and the range of working time with a computer. The test results showed a significant difference in the variable of employment status in a way that the individuals employed obtained higher scores than the unemployed ones. However, there were no significance differences between other aforementioned covariates and self-care indicators.

## Discussion

### Principal Findings

#### Impact of Diabetic Personal Health Records on Primary Outcome Measures

The test results demonstrated that the Web-based DPHR app had a positive impact on the primary outcome measure, namely in the status of self-care in general, and in 4 of its dimensions, in particular, including information of normal values, information of change trend, information of the latest measurement values, and information of date and time of the latest measurement values. Investigations show few studies on the relationship between PHR and self-care status. It is pointed out that in studies available in the field, the self-care index has been usually defined relatively homogeneous, but the important point is that any self-care index studied by the researchers may have different dimensions and questions, which can affect the efficiency of intervention. The self-care index assessed by the researchers in this study had 7 main dimensions and a total of 56 questions.

#### Impact of Diabetic Personal Health Records on Secondary Outcome Measures

Researchers have not found any positive effect between Web-based DPHR app and clinical outcomes including weight, HbA1c, serum creatinine, HDL, LDL, total cholesterol, and triglyceride. There are differences and sometimes contradictions among existing studies for the effect of PHR interventions on clinical outcomes associated with diabetics such as HbA1c and lipid profile. Most studies refer to a positive relationship of PHR [11,30,47,48], though several studies indicated no positive effect [30,49]. Researchers revealed that such contradictions could have several reasons, the most important ones being how to design the app, type of study design, duration of study, and study attrition rate. It is important to note that the impact of some of the positive studies is in doubt because of limitations and bias. Moderate to high risk of bias has been reported in 4 studies assessing the interventions [50]. It seems that relatively short-term duration of the study is the primary reason for the lack of positive association between DPHR and clinical outcomes in this study. Conducting a systematic review on the effectiveness of DPHR and clinical outcomes in patients with type 2 diabetes can be very useful.

#### Usability Evaluation of Diabetic Personal Health Records App

In this study, usability-testing process was carried out through a scientific process and with the participation of 20 specialists in medical informatics, 2 endocrinologists and 6 diabetics. The proper sample size is essential for usability testing, so that research, which found that up to 80% of usability has issues, can be determined with 5 to 8 participants [51]. Based on our usability testing, Jakob Nielsen's general principles for interaction design such as error prevention, consistency and standards, aesthetic and minimalist design, recognition rather than recall, and help and documentation are required to be considered, and which were addressed on our app through iterative refinement process [52]. One of the most important cases in intervention implementation is usability testing [53] but this is often neglected, with up to 60% of diabetes-related websites having a minimum of 4 usability errors. So it can be said that the gold standard for intervention development should be represented to obtain high intervention effectiveness [54].

#### Impact of Diabetic Personal Health Records on Visit Adherence

The results of Fisher’s exact test on the comparison of visit adherence scores revealed no significant difference in both intervention and control groups. Given the importance of disease follow-up and the treatment of diabetic patients by attending physicians, the patients paid attention to their visits, and their efforts in terms of planned visit adherence were implicit.

#### Impact of Diabetic Personal Health Records on Blood Sugar

The change trend of mean for FBS 2 hours after lunch and 2 hours after dinner exhibited that there was generally a sinus trend in all the above-mentioned cases. The main difference between this study and those in the related literature is that in our study we measured trends in blood sugar levels for 10 times, whereas such values were usually compared before and after intervention in most studies. In a study by Davies et al, by using DPHR for 6 months, blood sugar levels had improved in both groups, particularly in the intervention group [47].

#### Impact of Diabetic Personal Health Records on Blood Pressure

The change trend of mean for systolic and diastolic blood pressure in the intervention group revealed steady and stable trends, and no increasing or decreasing patterns were observed. Previous studies indicated no difference was noticed in the improvement of blood pressure in control and intervention groups [30,49]. No improvement was also found in levels of systolic blood pressure values in a study conducted by Dijkstra and only slight improvements were observed in diastolic values [55].

#### Correlation Between Diabetic Personal Health Records App and Covariates

The results of the chi-square test and ANOVA for analyzing the correlation between the trial covariates and the self-care index showed that there was a significant difference in the variable of employment status in a way that the individuals employed obtained higher scores than the unemployed ones. Moreover, in comparison with the previous studies, there was no significant difference in self-care index scores among type 2 diabetic patients in terms of their marital status, because more than 90% of the participants in this study were married. In a study by Bohanny et al, the scores of self-care behaviors were significantly higher in married individuals than those obtained by single people [56]. However, such conflicting results require more investigations.

### Strengths of Study

The strengths of our study are as follows: the evidence-based development process of the DPHR app (based on a systematic review), the inclusion of local experts’ opinions, and the iterative refinement of the app using the usability techniques. Both the value of evidence-based content development and the importance of usability testing in the app development process have been emphasized in several studies [53,54,57], pointing to the fact that such considerations have rarely been used in similar works [54].

### Limitations

A few limitations of our trial are as follows:

1. The primary need to recruit participants with minimum computer skills and Internet literacy was a limiting factor. Generally, in Web-based interventions, issues such as digital divide, computer literacy, age, and interest in technology can be effective in participant recruitment. The young, computer literates, and those having access to the Internet usually have a strong tendency toward participating in such studies. This trial is not an exception in principle. Such tendencies may present bias in our findings, and thus, our trial may not necessarily represent the actual distribution of the population being studied.

2. The trial sample only represents type 2 diabetes patients. It is possible that the findings could not be generalized to other types of diabetes disease.

3. Due to the nature of intervention, the investigators frequently requested the presence of the participants for the interviews. This induced discomfort to some of the participants. To address such issues, we provided financial incentives such as free visits and laboratory testing in order to encourage better involvement.

4. The other limitation of our study was the passive participation of some patients during the study, especially at the early stages. Therefore, lack of participation could lead to loss of useful information from patients. Therefore, reminders via telephone contact and SMS text messaging were employed.

5. Considering the limitations of our patient sample, the results should be interpreted cautiously [58]. The small sample size in our research may affect the representativeness and generalizability of the findings [59].

6. We concentrated on the comparison of primary and secondary outcome measures in our RCT study design, indicating explicitly a variation in consumer self-care status regarding the complexity of intervention [60].

### Implications and Future Directions

The methods and findings of this study are expected to be used as a suitable platform for other endocrine and metabolic disorders as well as other fields of medical science studies to assess the impact of the PHR intervention on the self-care index and clinical outcomes.

Multi-center study is proposed to be carried out on a broader level to ensure the effectiveness of the Web-based DPHR intervention on the self-care index and clinical outcomes. In this case, endocrinologists and patients with type 2 diabetes will be involved in the study in a wider range, which can be very important in the generalizability of the study findings, especially for developing countries. Moreover, the impact of DPHR efficiency on the level of decision-making of the endocrinologists is recommended to be evaluated through a proper RCT study.

Diabetes knowledge, involvement by health care providers, patient empowerment, and enhanced health care status will be promoted hopefully by the improved self-care status recommended by the Web-based DPHR to patients with type 2 diabetes mellitus.

### Conclusions

As a result of a systematic review of literature together with the representative sample of endocrinologists in Iran, a consensus was achieved on a Web-based DPHR model to improve self-care for type 2 diabetic patients. However, to take advantages of the DPHR, the given Web-based DPHR app was implemented and evaluated on type 2 diabetic patients after iterative refinement of the app user interface, using usability techniques. We found as a result of the Web-based DPHR app that the self-care scores in the intervention group were significantly higher than those of the control group. In total, we found no correlation between the DPHR app and covariates, including planned visit adherence, HbA1c, serum creatinine, HDL, LDL, total cholesterol, weight, the change trend of the mean blood glucose, and blood pressure.

## Abbreviations

ANOVA: analysis of variance
BMI: body mass index
DM: diabetes mellitus
DPHR: diabetic personal health records
EHR: electronic health record
FBS: fasting blood sugar
HbA _1c_: glycated hemoglobin
LDL: low-density lipoprotein
PHR: personal health record
RCT: randomized controlled trial
SMS: short message service
WHO: World Health Organization
